# Functional Connectivity Mapping in the Animal Model: Principles and Applications of Resting-State fMRI

**DOI:** 10.3389/fneur.2017.00200

**Published:** 2017-05-10

**Authors:** Martin Gorges, Francesco Roselli, Hans-Peter Müller, Albert C. Ludolph, Volker Rasche, Jan Kassubek

**Affiliations:** ^1^Department of Neurology, University of Ulm, Ulm, Germany; ^2^Department of Anatomy and Cell Biology, University of Ulm, Ulm, Germany; ^3^Core Facility Small Animal MRI, University of Ulm, Ulm, Germany

**Keywords:** translational MRI, *in vivo* animal model, neurodegeneration, connectome, mouse, rats, monkey, gene manipulation

## Abstract

“Resting-state” fMRI has substantially contributed to the understanding of human and non-human functional brain organization by the analysis of correlated patterns in spontaneous activity within dedicated brain systems. Spontaneous neural activity is indirectly measured from the blood oxygenation level-dependent signal as acquired by echo planar imaging, when subjects quietly “resting” in the scanner. Animal models including disease or knockout models allow a broad spectrum of experimental manipulations not applicable in humans. The non-invasive fMRI approach provides a promising tool for cross-species comparative investigations. This review focuses on the principles of “resting-state” functional connectivity analysis and its applications to living animals. The translational aspect from *in vivo* animal models toward clinical applications in humans is emphasized. We introduce the fMRI-based investigation of the non-human brain’s hemodynamics, the methodological issues in the data postprocessing, and the functional data interpretation from different abstraction levels. The longer term goal of integrating fMRI connectivity data with structural connectomes obtained with tracing and optical imaging approaches is presented and will allow the interrogation of fMRI data in terms of directional flow of information and may identify the structural underpinnings of observed functional connectivity patterns.

## Introduction

An unexpected observation in the noisy fMRI signal obtained from humans quietly “resting” in the absence of any specific task has ultimately led to the discovery of a phenomenon now well known as “intrinsic” or “resting-state” functional connectivity (1, 2). In 1995, Biswal and colleagues demonstrated that the apparent fMRI noise displayed coherent temporal patterns most prominently at low frequencies within anatomically distinct and spatially distributed neuron populations (3). Many efforts aimed at demonstrating that spontaneous fluctuations in the fMRI signal obtained at “rest” maintain meaningful functional activity (2, 4–6). Patterns of functional activity are topologically organized (7–9) within defined brain systems even across species (10), rather than representing artifactual byproducts of non-neurophysiological process including motion, cardiac, or respiratory factors. These landmark studies have received further overwhelming support from different approaches including positron emission tomography (11–13), magnetoencephalography (14), optical imaging (15), single-unit and local field potential recordings (16), and electroencephalography (17, 18) that systematically investigated ongoing spontaneous brain activity in relation to each other. Nowadays, the investigation of the spontaneous fMRI signal has grown into a major and rapidly expanding field for studying functional brain organization (19). In summary, *in vivo* imaging becomes an increasingly important phenotyping instrument in order to accelerate our understanding of the architecture in both healthy and diseased brains (20).

Besides the eponymous “resting” condition (3, 11), organized spontaneous activity in the fMRI signal has also been demonstrated under various states of consciousness including sleep (21, 22), anesthesia (23), coma, and minimally conscious state (24), in developing brain (18, 25), aging and brain maturation (26), and their potential genetic aspects (27, 28). A major compelling aspect of the fMRI signal has been the organized spontaneous brain activity across different species including mice (29–35), rats (29, 36–42), rabbits (43), dogs (44), and pigeons (45), with monkeys (21, 46–53), mice, and rats (54) representing the largest fraction of “resting-state” (rs-)fMRI investigations.

The purpose of this review is to introduce the principles of rs-fMRI with specification to animal measurements and applications to the animal model. We will address methodological issues from experimental design to rs-fMRI data acquisition. The focus will be on the emerging concept of translational imaging from *in vivo* animal models of brain diseases to clinical applications in humans that may eventually form the groundwork for fundamentally novel therapeutic approaches. The continuous and compelling engagement is highlighted in studying the functional connectivity patterns underlying cellular and molecular events in living animals since there are currently no sufficient *in vitro* or *in silico* models that can serve as alternatives to the use of *in vivo* animal models (55). This review will provide insights into the broad spectrum of data analysis and their interpretation at different abstraction levels, including voxel-wise statistics and the graph theoretical analysis of functional connectome organization.

## Exploring Functional Connectivity in the Living Animal

**Figure 1:**
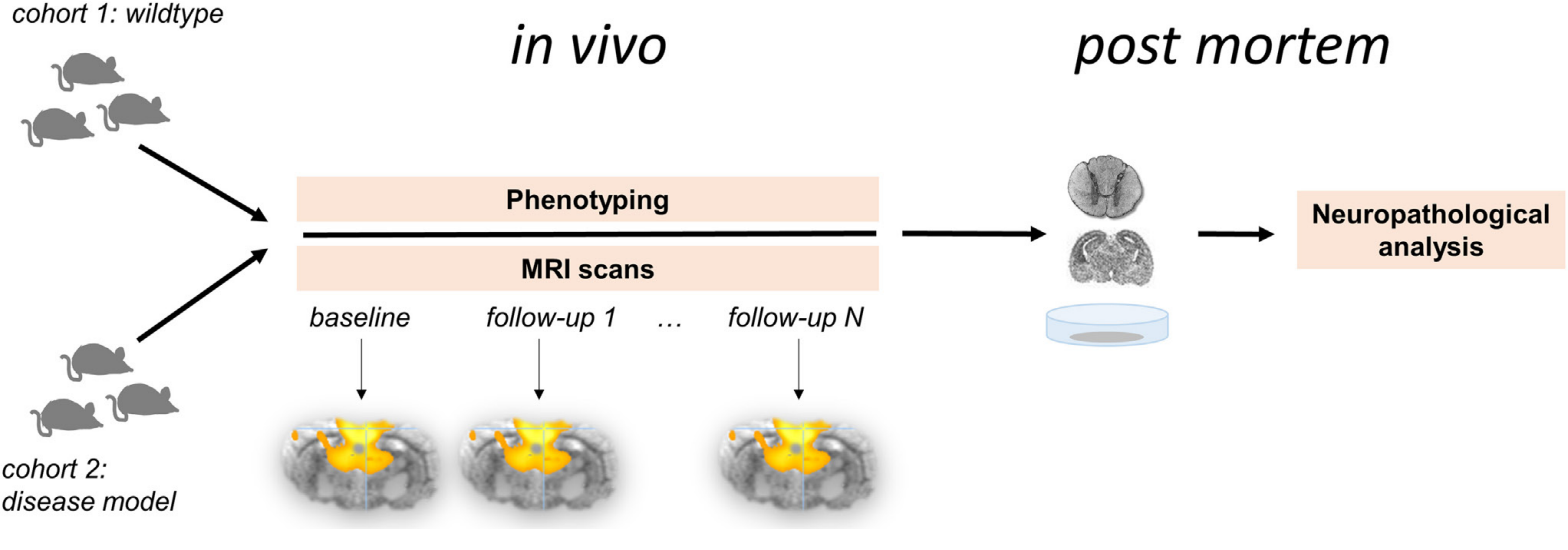
**Longitudinal multiparametric study concept for functional connectivity analysis in the animal model**. Two cohorts comprising wild-type animals (control group) and disease model undergo *in vivo* investigations followed by neuropathological analyses *post mortem*. This study design allows for the systematic analysis for the functional brain organization and its potential changes over time in association with phenotype-depending behavioral and histopathological parameters.

In contrast to the exponential growth of “resting-state” publications in humans (56), functional connectivity investigations of non-human species is considerably less often applied. Comparative “resting-state” fMRI studies in living animals will accelerate the ability to define and test translational animal models of brain pathophysiology (Figure 1). Altering the physiological condition of the animal model has been demonstrated to influence both brain “wiring” (57) and the patterns of functional connectivity (58). Humans and animals share similar features of functional brain network organization and support perceptual and cognitive core properties that are closely related to behavior (48, 59). Animal models allow genetic modification and can provide insights into the functional brain organization and the alterations in different disease models. Appropriate measures of functional connectivity may be used as surrogate markers or potentially biomarkers and may form a readout to validate animal models in conjunction with new therapeutic strategies (46).

### Neurophysiological Substrate

**Figure 2:**
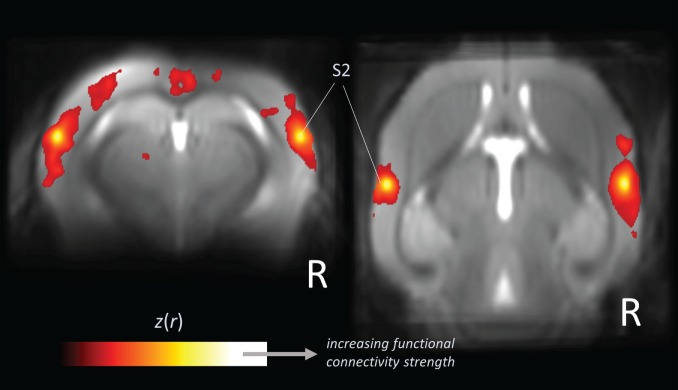
**Large-scale correlation patterns of the “resting-state” fMRI signal**. Data are shown from five wild-type mice acquired from a high-field (11.7 T) small animal MRI. Orthogonal brain section heat maps showing mean voxels for which the fMRI signal was correlated with bilateral seed regions in secondary somatosensory cortex (S2), which allows the identification of the somatosensory functional motor network. The *z*(*r*) values indicate the strength of correlation.

The functional networks revealed by rs-fMRI (Figure 2) provide a more general picture of brain function across species. Brain regions that are coactive during specific tasks (as measured by “task-based” fMRI) tend to be integrated within corresponding functional networks (as obtained by rs-fMRI) (9). This groundbreaking observation in more than 30,000 fMRI data sets by Smith and coworkers (9) allow the conclusion that task-induced activation maps underestimate the size and number of functionally coupled neuron populations (1). Many suggestions have emerged explaining the underlying mechanisms which link neural activity to hemodynamic responses. There are proposals of rehearsal, learning consolidation, future preparation (60), and variations of neuronal excitability (61), but there is not yet conclusive evidence for any of these concepts. Factors such as anesthesia influence the shape of functional connectivity patterns (62); however, the well-defined networks including the default mode (32, 63) remain stable across species and its integrity and topological principles remain preserved as demonstrated for widely dissimilar physiologic states in rats (64).

A further indirect conclusion about the fundamental role of the brains’ ongoing activity in (nearby) all species can be drawn from a metabolic perspective since ongoing activity is energetically costly (2) while brain organization across species is economically optimized (65). This statement is in agreement with the fact that the mammalian brain has maintained the hierarchy of brain oscillations, irrespective of the species’ brain size (66). Brain oscillations are topologically organized across species which is in line with evolutionary processes (10). Moreover, it appears save to assume that low frequency correlations are a general feature of neural systems (67). Currently, the understanding of ongoing spontaneous activity and its neurophysiological substrate is still under investigation (68).

### Animal Models

Animal research has a pivotal role in neuroscience and has substantially contributed to our understanding of various conditions and diseases. The benefits of animal research have been tremendous and have substantially contributed to ongoing advancement of medicine and neuroscience as well as the health of both human and animals (69). Each experiment in living animals has to be designed within a strict ethical framework. The beneficial effects on humans have been proven, even though scientists are encouraged to further improve better public understanding and to emphasize implications on humans (55).

Comparative conclusions from cross-species functional connectivity investigations argue that the functional interaction of brain networks support the broad variety of different cognitive processes and provide essential features for survival (46). A large body of evidence attributed abnormal functional connectivity patterns to multiple psychiatric, neurological (70), and developmental disorders (71). Since advanced genetic techniques allow manipulation of the genome and precise control of gene expression in rodents, transgenic models of human neuropathology are becoming increasingly important. One challenge is to differentiate between normal aging and the onset of a pathological neurodegenerative processes. For neurodegenerative diseases including Alzheimer’s disease, Parkinson’s disease, and amyotrophic lateral sclerosis, it is known and broadly accepted that the underlying pathological process in human propagates in a characteristic fashion and can be attributed to different subclinical and clinical disease stages (72). Animal models of functional brain organization at different neurodegenerative disease stages may allow to define possible readouts for possible surrogate markers and enhanced drug trials. Transgenic mouse models of neurodegenerative pathology revealed abnormal functional connectivity patterns in agreement with findings in humans (54). In particular, these findings resulted from studies in cholesterol transporter apolipoprotein ε (ApoE) mutant mice, a genotype associated with an increased risk to develop neurodegeneration (73). In ApoE4 mice mutants, functional connectivity has been successfully used as a readout to evaluate dietary treatment that improved brain connectivity (74). A role for microglia in promoting the maturation of circuit connectivity during development has been demonstrated in Cx3cr1^KO^ mice compared to wild-type animals by using functional connectivity measures as a readout for neurodevelopmental impairment (71). This study addressed the gap between neurochemical mechanisms of microglia-mediated synaptic pruning und functional connectivity. Many other studies in transgenic models such as autism (75) reported abnormal functional connectivity in association with a phenotype. Standardized brain trauma models in animals have not been established yet. Studying functional alterations associated with traumatic injuries (76) under controlled conditions in the animal model may gain detailed insights into altered patterns of functional connectivity and the effects after recovery. We can hypothesize that the functional brain network is regionally damaged and this lesion may trigger functional reorganization as a compensatory response. The impact and its location can be controlled together with the phenotype (transgenic mice models exist that are defined by the responsibility and resistance against traumatic brain injuries) in animal experiments (77), which is not possible in humans. Functional (re-)organization in association with a behavioral correlate could be a valuable readout for the investigation of traumatic injuries.

Thus, as long as we do not have a full brain model proven to provide all complex neural and neurochemical properties necessary for modeling brain dysfunctions and therapeutical concepts for different phenotypes, there will be the need for animal models as a biological system capturing the complex humans’ overall brain architecture as closely as possible. Medical advances of the past decades underpin their role in the development of therapeutic concepts that may eventually pave the way for causal treatments of neurodegenerative diseases. Many studies reported altered functional architecture of intrinsic activity in neurological and psychiatric disorders (78); hence, here we review respective studies on translational animals utilizing rs-fMRI. Animal models encompassing disease or knockout models allow for a great range of experimental manipulations which are not applicable to humans. Moreover, postmortem validation of the underlying pathological process—which is very limited in human studies—is easier to achieve in animal studies. Most studies of functional connectivity utilize the non-invasive fMRI approach because it provides a promising tool for cross-species comparative approaches.

## Principles of “Resting-State” fMRI Data Acquisition

In humans, “resting-state” data are easy-to-acquire by typically asking subjects to “rest” quietly with their eyes closed and motionless in the MRI scanner for about 5 min; indeed, “rest” is poorly defined and has been attributed to a control condition in the context of a commonly used block design in “task-based” fMRI studies. The definition of “rest” (or better “task-free” condition) becomes even more complicated and nearby impossible to control for in the context of non-human rs-fMRI. The vast majority of rs-fMRI investigations in animals including small animals require anesthesia which minimizes motion artifacts, physiological stress, and training requirements (46). Motion corruption is a critical issue due to its high susceptibility during fMRI data acquisition (19). Despite the limitations of controlling the “rest” condition and the various types of anesthesia and consciousness states, the functional data are largely comparable even across species.

The “resting-state” fMRI sequence is a ${\text{T}}_{2}^{*}$-weighted signal that is sensitive for neurally driven changes of the oxy-to-deoxyhemoglobin ratio, i.e., blood oxygenation level dependent (BOLD), which changes the magnetic properties (79). The resulting fMRI data set consists of a series of echo planar images, i.e., volumes that are equidistantly obtained during the scan. The voxel size is identical for each volume and defines the spatial resolution; the time needed to record each volume (repetition time) defines the temporal resolution (79). Table 1 provides representative examples of commonly used scan parameters for rs-fMRI data acquisition in key laboratory animals. The voxel size varies across species depending on the MRI scanner, whereas the typical number of voxels of a brain is relatively constant across species. For instance, a typical voxel size for whole-brain scans of an 11.7-T ultra-high-field MRI used to scan mice is about 150 μm × 150 μm in-plane resolution and 250–500 µm slice thickness; in comparison, a typical spatial resolution of a state-of-the-art 3 T for humans is about 2 mm × 2 mm and slice thickness of 1.5–3 mm. Smaller voxel sizes allow a better separation of different tissue types at the expense of adverse signal-to-noise ratio and decreased temporal resolution (a volume takes longer to acquire). Larger voxel sizes covering different tissue types may contain shared hemodynamic activity from different neuronal populations—a phenomenon called partial volume effects. The temporal resolution is typically around 2 s, which is appropriate to measure slow-frequency fluctuations (<0.1 Hz), i.e., infra-slow waves (0.01 < *f* < 0.10 Hz) (21), as it is intended by “resting-state” fMRI. However, a time repetition is associated with limitations arising from the Nyquist–Shannon “sampling theorem”—a fundamental law of digital signal processing: the frequency band being investigated is limited to 0 < *f* < 1/(2 × TR). That means, for TR = 2 s is the highest frequency 0.25 Hz and all frequencies >0.25 Hz (e.g., cardiac pulse of about 1 Hz = 60/min) are confounding the signal, i.e., aliased, and cannot be filtered. Hence, other frequency bands such as the typical EEG bands ranging from slow-frequency (0.1–1.0 Hz) and delta (1–4 Hz) (80) to gamma (25–100 Hz) (81) cannot be investigated using “resting-state” fMRI. Efforts have been made and related research continuous to optimize both spatial and temporal resolution by using acceleration techniques to permit more rapid whole-brain-based volume acquisition (82).

**Table 1:** **State-of-the-art “resting-state” (rs-)fMRI protocols for key laboratory animals compared with humans**.

	Resting-state (rs-)fMRI (mice) (71)	rs-fMRI (rats) (35)	rs-fMRI (monkeys) (83)	rs-fMRI (humans) (84)
Field strength (T)	7.0	9.4	7.0	3.0
Slices, *n*	16	12	30	47
Slice thickness (mm)	0.75	1.00	1.50	3.00
Voxel size (mm)	0.23 × 0.23 × 0.75	0.23 × 0.23 × 1.00	1.30 × 1.30 × 1.50	3.00 × 3.00 × 3.00
TR (ms)	1,000	2,000	2,000	3,000
TE (ms)	15	16	16	30
FOV (mm)	23 × 20 × 12	30 × 30 × 12	96 × 96 × 45	216 × 216 × 216
Volumes, *n*	360	150	300	124

*Depicted are representative scan parameters for whole-brain rs-fMRI data acquisition using echo planar imaging (EPI, $T_{2}^{*}$)*.

## Analysis of Spontaneous Neural Activity from the fMRI Signal

Although the rs-fMRI data acquisition is relatively easy even in the living animal, the analysis is not. Several major denoising approaches have been suggested to provide an optimal estimate of the neurally driven variance in the “resting-state” fMRI signal. The measured signal is considerably confounded by non-neural signals including motion artifacts, respiration, cardiac pulse, and their variations over time such that only about 4% of the total variance [for high quality data of the human connectome project (85)] in the fMRI signal accounts for neurally driven signals (86). Hence, the vast majority of the preprocessing pipeline of the fMRI signal copes with denoising of the data, i.e., extracting the neurally driven fMRI signal.

### Preprocessing “Resting-State” fMRI Data

Preprocessing typically includes motion correction, spatial smoothing, temporal demeaning, detrending and band-pass filtering, regression of nuisance covariates, and normalization into a common stereotaxic space (87). These steps are commonly used across species (32, 71, 88) and are briefly explained. Nearby all fMRI studies correct for motion artifacts that occur even under anesthesia due to respiration, cardiac pulse, and muscle relaxation. Head motion can lead to severe image degradation (89) and can result in false-positive functional connectivity (19). Motion correction can be done by computing an estimate of each volume with respect to a reference volume (e.g., the first volume) for all degrees of freedom (*x, y, z*, pitch, roll, yaw), which allows for a rigid brain transformation (90). Some investigators suggested to withhold motion-contaminated images from analysis and provided elaborated approaches that model motion influences to the fMRI signal (89, 91). Spatial filtering is applied to each of the volumes by typically using a Gaussian blur filter (3-D bell shape representing normal distribution) in order to improve the signal-to-noise ratio at the expense of “blurring” the images, i.e., decreasing spatial resolution, according to the “matched filter” design. The filter parameters are given as the full-width at half maximum (FWHM) for each spatial dimension (*x, y, z*); the filter length of twice the voxel size in dimension given by the scanner protocol is a common choice, e.g., for a (hypothetically) isotropic (400 µm)^3^ voxel size of a small animal MRI a good choice would be a Gaussian filter with 800 µm FWHM in each dimension (92).

Possible scanner drifts during the rs-fMRI data acquisition should be voxel-wise removed along each time series by linear detrending (93). Linear detrending can be performed by subtracting the linear fit of the voxel time-course. The frequency spectrum of the time series for each voxel is commonly band-pass filtered using cutoff frequencies in the range of about 0.01 < *f* < 0.1 Hz (70), some investigators perform low-pass filtering (*f* < 0.1 Hz). Temporal filtering is required in order to limit the frequency spectrum to the neurophysiologically interesting infra-slow waves that are termed the spontaneous low frequency fluctuations. Usually, the first volumes (about 10 volumes) are discarded from further analysis due to the transient temporal filter response (87) and to allow the subject to adapted to the experimental condition (94), for instance, relaxation and minor changes in head position due respiratory or cardiac factors may likely occur at the beginning of an experiment even under anesthesia.

Aiming at further denoising, most rs-fMRI investigators remove various nuisance covariates by using non-physiological signals as a regressor in a general linear model (95). These nuisance regressors include motion estimates and tissue-based regressors; in addition, the derivatives and backward differences can be used. Tissue-based regressors can be computed from the averaged signal across voxels form a ventricle mask, from the cerebrospinal fluid signal, from a white matter mask, from a whole-brain mask, and from the signal of the skull or background clutter (91). In summary, preprocessing is mandatory and can substantially improve the data quality in animal MRI scanning, although *post hoc* rs-fMRI denoising and artifact reduction remains challenging. The discussed preprocessing steps are similar for human and data from living animals.

There are numerous techniques available for denoising the rs-fMRI signal, but there is no gold-standard yet. The performance of these techniques depends on the preprocessing steps utilized and its order (96). A standard preprocessing pipeline can include any of the following steps: motion correction, resampling, removing of non-physiological signals, nuisance regression, temporal filtering, and spatial smoothing. Each of these steps requires a set of parameters. The steps and their order of the preprocessing pipeline should be carefully selected with respect to the MR acquisition parameters, species-specific traits, and the overall aim of the investigation. Table 2 provides a comprehensive summary of commonly used techniques in recent animal rs-fMRI investigations.

**Table 2:** **Frequently used preprocessing steps in the rs-fMRI data analysis pipeline (including denoising) according to recent animal studies, e.g., Ref. (43, 71, 97–99)**.

	Description	Possible drawbacks	Typical values
Head motion correction	Reduces the potential influence of head motion (which is also present in anesthetized animal)	Partial volume effects	All six degrees of freedom
Resampling	Provides data in a common grid with user-defined voxel sizes (which is particularly interesting for merging protocols)	Partial volume effects	Cubic grid, size depends on the species and overall aim of the rs-fMRI investigation
Regression of nuisance covariates: global signal	Reduces linear and non-linear dependence of signals that are assumed to represent no useful physiological information	Removal of superimposed neural signals	Motion estimates, white matter, CSF, global signal
Temporal filtering	Attenuates non-physiological frequencies and restrict the signal to the infra-slow wave spectrum	Attenuation of physiological frequencies around the cut-off frequencies	0.01 Hz < *f* < 0.08 Hz
Spatial smoothing	Increases signal-to-noise ratio by reducing uncorrelated noise	Blurred spatial resolution	Two times the native spatial resolution according to the rs-fMRI protocol
Discarding volumes	Removes transient temporal filter response and scanner oscillation at the beginning; allows the subject to adapted to the condition	Reduction of number of volumes	10–15
Fisher’s *r*-to-*z* transformation	Improves normality of correlation coefficients	Non-linear transformation	–

### Providing Data in a Common Stereotaxic Space

For data analyses across multiple MRI scans from different animals (of a given species), it is essential to align the acquired echo planar images into a common anatomical space in order to have a clear concept of localization in the brain. That means the spatially matched (i.e., “registered”) images should ideally provide the same spatial location in each subject. Good spatial normalization is a challenging procedure given that the brain’s complex cortical folding differs dramatically between individuals even of the same species (100). Investigators of human studies typically transform the images into the Montreal Neurological Institute (MNI) stereotaxic standard space (101); common practices are linear and non-linear (non-affine) registration algorithms (102). For animal MRI data, no such commonly accepted stereotaxic standard space has yet emerged, even not for key laboratory animals. However, neuroanatomical information for the adult and developing brain in mice, humans, and other non-human primate from the online public resource Allen Brain Atlas (103) can be used as a template in standardized coordinates. The Allen Mouse brain atlas, for instance, includes a full-color, high-resolution anatomic reference atlas accompanied by a systematic, hierarchically organized taxonomy of mouse brain structures that can be used as a basis for spatial normalization. A wiring diagram of the whole mouse brain at a mesoscale has been defined using standardized labeling, tracing, and imaging of axonal connections (104). Although the mesoscale structural connectome definition of the mouse has received great support (105), a functional parcellation of the mouse brain has not been presented, so far. For rats, a stereotaxic MRI template set for the rat brain with tissue class distribution has been published (106).

Normalization is generally achieved by co-registering all images from a study to a common echo planar image template (101). Different algorithms can be utilized to warp the individual images on the common template (107, 108). These commonly applied algorithms use a multiparametric affine transformation followed by non-linear mean squared difference matching. The normalization approach is principally similar for animal MRI data and humans and the quality of normalization into a common space mainly depends on the used template. The template image used could be one predefined template such as MNI template for humans (109) or a study-specific template can be created by averaging across a number of different subjects (e.g., whole study population, sub-cohort regarding phenotype, age, scanner protocol) that have been transformed in a common space (110). Customized study-specific templates in a common stereotaxic space are highly recommended for registrations purposes (111) because inter-subject normalization within the study cohort, for instance, with respect to global brain atrophy (e.g., in neurodegenerative disease models), is considerably improved. State-of-the-art animal fMRI analyses techniques (71) used a digitalized version of a mouse brain atlas utilized to create a template for fMRI data analysis. For instance, “resting-state” in dogs (44), a rarely investigated species for which no well-accepted brain atlas in a common space exist, used the functional image of one dog as the template on which the other dog was matched on. Normalization without using a common template can be performed by defining landmarks that allow a transformation according to the coordinates of these landmarks. Müller et al. (108) has suggest a landmark-based normalization procedure prior to a registration to a common template by manually defining eight landmarks in the first volume for each rodent (57, 112), a procedure adapted from human studies. The normalization procedure is typically refined by non-linear normalization of the individual echo planar images onto the study-specific template following the basic ideas of Ashburner and Friston (107) of minimizing the squared differences of regional intensities. In summary, the quality of normalization determines the ability to compare results between subjects or groups. At least small errors are inherent to any normalization procedure and differences in individual brain anatomy including the complex cortical convolution cannot be completely overcome by normalization into a common stereotaxic space.

### Functional Connectivity-Based Brain Parcellation

For spatial normalization, (study-specific) templates are required. Templates commonly refer to atlas-based definitions of the respective species (103), but the underlying brain structure does not adequately capture functionally segregated modules, i.e., “nodes” in the graph theoretical context, because it involves the risk of mixing different time courses within a single node (113) The definition of nodes across the whole brain is challenging but mandatory for robust and reliable functional connectivity analyses. Data-driven approaches including independent component analysis (114) have been introduced for brain parcellation and for delineating resting-state functional networks in humans (115) and rodent (32). Graph theory-based approaches provide another data-driven approach for functional brain network identification without the need for *a priori* information (116). A data-driven whole-brain functional parcellation algorithm that jointly optimizes the group and the individual parcellation has been introduced for the human brain (113, 117). The resulting functional brain atlas has been released (113), and it comprises up to approximately 300 functionally homogeneous subunits. The application of this data-driven procedure to rs-fMRI data of animals could be promising for studying functional brain organization more accurately, which is mainly important when behavioral differences between groups or across time are being analyzed.

### Functional Connectivity Measures

**Figure 3:**
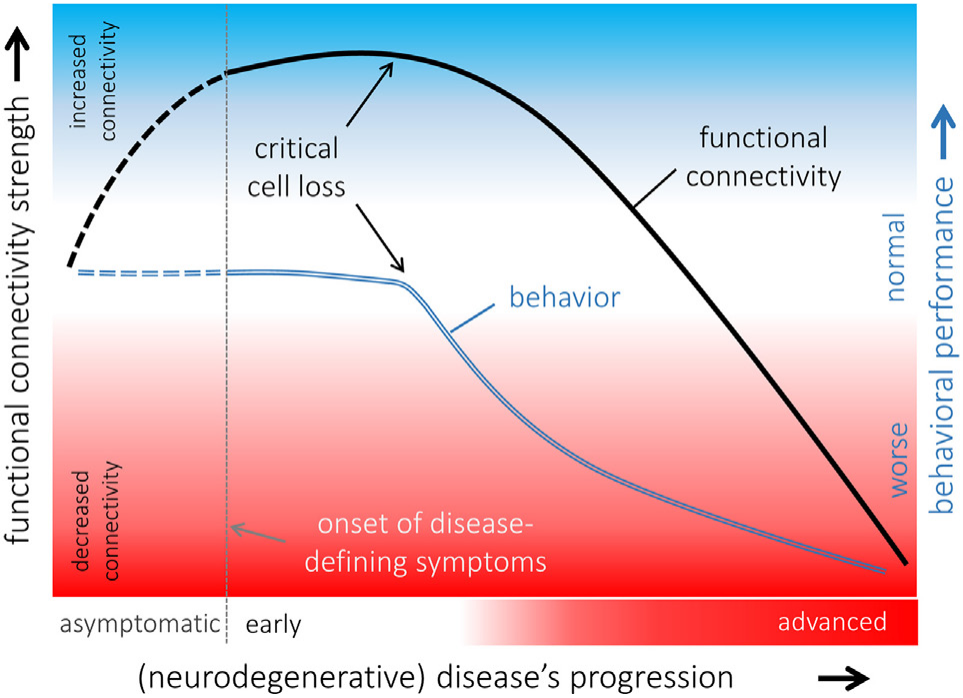
**Hypothetical model of functional connectivity alterations in association with behavioral performance in the course of neurodegeneration**. The pattern of functional connectivity changes (black line) and its association with behavior (blue line) indicated that functional connectivity increases in a potentially compensatory response to ongoing cell degeneration in order to maintain “normal” behavioral performance as long as possible. When a critical cell loss is reached, i.e., the functional reserves are exhausted, behavioral performance declines, and functional connectivity decreases upon a disconnection syndrome with poor behavioral performances presented in an advanced disease state. It remains open whether functional connectivity is already altered in an asymptomatic phase of an underlying neurodegenerative pathological process (dashed lines).

After the rs-fMRI data have undergone steps of preprocessing, a broad spectrum of functional connectivity analysis methods including measures of coherences, frequency spectral analysis, dynamics, and causal influences among time series is available (118). The simplest measure of signal similarity is the Pearson correlation coefficient which is widely used to measure the strength of pairwise region-to-region connectivity of the slow BOLD fluctuations and which allows for the definition of functional connectivity networks (Figure 3). The specification of regions of interests (also termed “seeds”) depends on the study design and is by definition a hypothesis-driven approach (119), in contrast to data-driven independent component analysis (mentioned as a tool for denoising), a mathematically sophisticated signal decomposition technique that allows to separate various “independent” sources (114). Both techniques have been successfully applied to investigate functional connectivity organization in human and living animals including rodents (32, 71).

The set of pairwise region-to-region connectivity measures (e.g., Pearson’s correlations) obtained from a fine-grain voxel-wise parcellation of the brain can be used for graph theoretic analysis at a higher abstraction level (120). A brain’s functional organization has features of complex networks at the whole-brain scale and the cellular level (121). Connectome-related differences in brains across species follow an allometric scaling relation across the entire spectrum of simpler to higher order species (10). Features such as highly connected neuron populations (“hubs”) (122), modularity (116), rich-club organization (123), and efficient small-world topology (70) can be systematically quantified by means of graph theory (124) across species.

Recent advances in mathematical tools to infer connectivity and to estimate the connectivity strength have been made, which are particularly useful in small samples as it is the case for most animal rs-fMRI studies. Graphical models that allow to reach reliable estimation of the inverse covariance even when the sample size is close to or even less than the number of brain regions investigated have been demonstrated in positron emission tomography-based connectivity analysis to be promising candidates for rs-fMRI investigations (125). Moreover, graphical models allow for graph-based analysis and have been proven to be used in a discriminative way in an unsupervised classification framework (126). These approaches in pattern classification and unsupervised learning algorithms allow to separate groups, e.g., with respect to the disease model, on the basis of features extracted from imaging data as proven in human studies (127, 128).

### Longitudinal Data

The conceptual design of multiparametric studies (as illustrated in Figure 1) highlights the increasing importance of longitudinal measurements (129). Follow-up data enable to investigate the intra-subject rate of functional connectivity changes over time, which is especially useful when studying developmental processes or tracing the course of disease propagation (98). Information on intra-subject changes over time additionally allow to longitudinally compare groups with each other in order to investigate whether multiple groups, e.g., different phenotypes, change over time with respect to functional brain connectivity alterations across a time period of interest. Longitudinal studies in animals, in particular in rodents, are of special interest since the study can be designed across the entire lifespan in contrast to humans where longitudinal studies have to be designed for many years or even decades to capture a sizeable part of the human lifespan.

## Interpretation of Functional Connectivity Data

At the very basic level, strong patterns of low-frequency BOLD coherence among functionally related brain areas creates strong suspicion that two (anatomically) distinct brain regions are neurally communicating in an organized fashion. The brain’s many regions—in humans and living animals—appear to functionally interact with each other in the “resting” condition (130) and even when a subject (24) or an animal is under anesthesia (53). The very nature of the underlying neurophysiological mechanism still remains not fully explained; however, many conclusions can be drawn about the functional brain organization. In what follows sheds light on commonly observed rs-fMRI patterns in healthy and diseased brains and their possible interpretation.

### “To Rise and to Fall”: On the Meaning of Altered Functional Connectivity

It is safe to assume that each deviation from “normal” connectivity represents an abnormal possibly pathological condition of the brain (131), although the very nature of the underlying ongoing BOLD fluctuations under diverse conditions ranging from “rest” to deep anesthesia across species is not fully understood. Functional connectivity can alter in both directions defining decreased (hypo-) and increased (hyperconnectivity) conditions, as observed from “seed-based” correlation analysis.

It is virtually straightforward to explain decreased functional connectivity that may eventually lead to a full disconnection syndrome in the context of cell degeneration or impaired functioning of segregated brain modules. Disrupted functional integration with decreased connectivity is associated with cognitive deficits (132) and poor behavioral performance as demonstrated in transgenic mice (71).

By contrast, increased functional connectivity, i.e., stronger BOLD synchronization, is more challenging to interpret; increased functional connectivity has been demonstrated in various neurodegenerative conditions in human studies (133–137) and in rats (138, 139). Increased functional connectivity is often interpreted as an adaptive response to an underlying pathological process that could be explained by recruiting additional “resources” by functional reorganization in order to compensate for neural malfunctions and to maintain cognitive and physical performance (136). This model is supported by rs-fMRI investigations in rats following neuropathic injury (138) where functional reorganization including patterns of increased functional connectivity has been demonstrated. At a clinical level, in patients with Parkinson’s disease, who exhibit a neuropsychologically confirmed “normal” cognitive performance, the observed patterns of increased functional connectivity across cortical functional networks in comparison with age-matched healthy controls are likely attributed to functional “compensatory” reorganization (135) in general agreement with finding in rats. There is growing evidence that increased functional connectivity can be the first state of abnormal brain functioning in neurological conditions including epilepsy as demonstrated for rats (139) and in the course of neurodegenerative diseases (131, 135). Given that functional connectivity is increased in an early state of the disease and a disconnection syndrome is present in an advanced state of the disease due to ongoing cell damage, a hypothetical model of functional connectivity alterations in association with behavioral performance can be proposed (Figure 3): functional connectivity increases upon the neural “reserves” are exhausted while maintaining “normal” behavior. Due to ongoing cell loss (e.g., neurodegenerative disease) or focal damage of respective functional modules (e.g., traumatic brain injury), core nodes of the respective functional connectivity networks decreases functional connectivity with other nodes of the networks and become eventually functionally disconnected in case of ongoing cell loss without remission (137). The likely transient state from patterns of increased to decreased functional connectivity may be accompanied by behavioral and cognitive decline. There appears to be increased functional coupling in the asymptomatic phase of neurodegenerative diseases consistent with our model as supported by a recent study on subjects at genetic risk (140). This further calls transgenic animal models to validate the suggested model of functional alterations in the course of a disease and animal studies with prospective to define abnormal patterns of functional connectivity prior to disease onset are required.

Another explanation for increased functional connectivity could be the loss of the inhibitory influence that may lead to pathological firing associated with abnormally increased functional connectivity (133). This would mean that brain regions excessively firing in a coherent fashion are no longer able to both share “useful” information with each or to interact with other functional modules (87, 137). Animal models are needed to challenge this hypothesis and to investigate the pathophysiology of increased functional connectivity in more detail.

### Connectomics—Functional Connectivity from a Network Perspective

The brain is an efficient representation of a complex system (65, 141) and has remarkable properties across species. It consists of spatially distributed and functionally specialized regions that continuously share information with each other (70). Graph theoretical approaches for the analysis of functional networks provide a powerful way of quantifying properties of the brain’s functional system (121). A functional network is defined in graph theory as a set of nodes, i.e., functionally segregated brain regions, and edges, i.e., a functional connectivity measure, between two nodes (123). The definition of nodes is a critical factor as discussed in the context of (functional) brain parcellation. Given that a set of a sufficient number of nodes has been defined, graph topology can be quantitatively described by a broad spectrum of network measures. An association matrix of functional connectivity measures form the basis for a graph analysis (142) and contains pairwise measures of functional connectivity, typically Pearson’s correlation coefficient of slow BOLD fluctuations in both nodes. Many measures of useful properties that characterize the functional network organization can be computed including basic concepts, measures of segregation, integration, motifs, and resilience, and other concepts such as “network small-worldness” (124). The graph theoretical measure that is going to be applied should be guided according to a clear hypothesis and is fully appropriate for functional connectivity investigations in the animal model. For instance, a graph theoretical approach has been recently applied for the investigation of functional connectivity in rats prior and after stressful event exposure (143) and in rats following neuropathic injury (138). The basic concept of graph-based connectomic analysis between the animals’ and the humans’ brain are nearby identical, and it has been demonstrated that fundamental properties of brain topology in rats are conserved in the same manner as in the human brain (38). In summary, modeling the functional brain connectivity as a graph, with nodes being segregated functional modules and edges being functional “region-to-region” connectivity strengths, opens a new avenue for investigating functional brain organization in multiple neurological and psychiatric conditions in a translational framework.

### Limitations and Pitfalls

The presence of noise and artifacts limits the study of functional brain organization and inadequate removal of artifact can fundamentally change the conclusions of each and every rs-fMRI study. However, a perfect separation between neurally driven BOLD activity and noise is practically impossible (144), despite the many advanced and continuing efforts being made for denoising the fMRI signal (145). Thus, it is crucial to interpret the outcomes with caution. The nature of BOLD fluctuations and the temporally correlated characteristics are an indirect measure for functional connectivity and is technically constrained by the poor signal-to-noise ratio and limited spatial resolution (146) although many advances in scanner technology, image acquisition protocols, and experimental design have emerged (79). The spatial and temporal limitations of the “noisy” BOLD signal could not be fully overcome by the standardized preprocessing pipeline for fMRI data (147) and conclusions drawn should always incorporate the given limitations of the methodology. However, improvements of analysis techniques have considerably helped to improve the results toward a more reliable interpretation that always have to be seen in a context with the conclusions drawn from the functional MRI data across species.

Various states of consciousness and possible drug administration cannot be (fully) disentangled from “normal” variability of patterns in functional brain connectivity. Many factors dynamically confound brain functioning, and the challenge remains to define robust measures that allow for the definition of an abnormal or pathological state. Caution must be taken when interpreting functional connectivity data since network measures were bound to be unstable across thresholds, i.e., thresholds that defined whether two voxels are functionally connected with each other (148). This methodological limitation has been overcome by an elaborated connectome-based functional connectivity analysis based on a data-driven graph-based parcellation analysis (113) such that functional connectivity profiles act as a “fingerprint” and are both robust and reliable across subjects (149). Comparing groups that may exhibit systematic differences, e.g., motion, can confound the results, e.g., in disease models of movement disorders.

The contribution of inhibitory and excitatory neuronal coupling to functional connectivity measures cannot be disentangled (8). The by-default limited spatial resolution of “resting-state” data and the limited signal-to-noise ratio of the BOLD signal make spatial smoothing a necessary preprocessing analysis step, in order to increase the signal-to-noise ratio, at the expense of partially mixing the signal between gray matter and the neighboring white matter. Hence, the clusters cannot be fully disentangled between gray and white matter. The animals’ head motion-induced artifacts—even present under anesthesia—might contribute to the rs-fMRI signal and are believed to produce spurious correlation patterns (19), which should be considered in any interpretation of the results.

### Toward the Integration of Structural Connectomes and fMRI Functional Connectivity

In the last few years, large-scale mapping of physical connectivity in the rodent brain has become feasible, thanks to the convergence of viral and non-viral tracing approaches with high-throughput technologies for image acquisition and image reconstruction. The Allen Mouse Brain Connectivity Atlas (104) initiative has used adeno-associated virus to express green fluorescent protein systematically across a large number of cortical and subcortical structures and then, using a dedicated imaging technique based on serial sectioning and two-photon imaging, has produced imaging datasets spanning the whole mouse brain for each of the injection. Once reconstructed, the resulting database has provided mesoscale connectivity down to cellular level. An independent effort, based on non-viral tracing systems (150), has provided a comparable connectome map with mesoscale resolution in which cortical subnetworks could be identified. Additional connectome datasets with cellular resolution have been obtained for specific subnetworks such as basal ganglia (151). These structural information provide a conceptual framework for the interpretation of functional connectivity data by allowing the selection of subnetworks, to be explored in fMRI data, based on the known directionality of connections between areas. However, each node of the mesoscale maps is populated by multiple local microcircuits endowed with both long- and short-range connectivity. While this subpopulation of neurons may not be directly detectable by fMRI data, they may have a significant footprint in terms of brain-wide activity. These subnetworks are now also amenable for investigation and manipulation: the use of rabies vectors (152) allows retrograde monosynaptically connected neuronal networks to be imaged at very high resolution [e.g., Ref. (153)] and directionality of connectivity can be used to complement fMRI information (e.g., by reconstructing all synaptically connected input to a given structure). In other terms, once the spreading of functional events within networks is observed, the structural datasets either at mesoscale or at single-cell resolution allow their direct mechanistic interpretations in terms of the simplest neuronal ensembles involved in the generation of the observed functional patterns.

In particular, the directionality of connectivity is, theoretically, easy to determine in structural connectomes since the origin of the axons and their destination can be distinguished; however, the existence of dense networks of reciprocal connectivity [in cortico-cortical or cortico-subcortical loops (104)] may require additional experimental strategies (e.g., *in vivo* silencing *via* chemio- or optogenetics) to determine the causal chain of connectivity.

Mesoscale connectomes obtained with current optical imaging technologies may offer a significant resolution advantage compared to MRI techniques (single axons can be individually resolved) although the size of the source volume (e.g., the volume of the injection site) encompasses several whole cortical columns; however, the structural connectomes do not offer a dynamic image of the connectivity but only a static picture of the averaged connectivity diagram. Furthermore, structural data do not hint at the relative functional importance of connectivity: a small number of axons from modulatory subcortical structures may have a much larger impact on the dynamic organization of functional networks than large number of intracortical connectivity.

In order to achieve the most effective combination of high-resolution connectomes and fMRI connectivity maps, the integration of datasets obtained with largely distinct technological platforms will become necessary, including approaches for co-registering datasets with very different resolution. Ideally, the use of light-sheet microscopes may allow the coregistration of the same brain samples first in fMRI *in vivo* and thereafter at single-cell resolution *ex vivo*, allowing functional–structural connectivity to be correlated at single individual level.

## Concluding Remarks

“Resting-state” functional connectivity analysis is a rapidly expanding approach to study the functional brain organization and has emerged as a major area of neuroimaging in both humans and animals. The emerging view that intrinsic activity might provide a more complete picture of brain function than task-induced activity has opened new pathways and allows for functional connectivity investigations even in anesthetized animals. Extending the use of rs-fMRI investigations in various types of clinical conditions using appropriate animal models is a promising avenue of ongoing research that may help to form a groundwork for understanding the fundamental properties of the functional organization in such models in a unique way (105, 150). Continuing research of functional organization in key laboratory animals (e.g., macaque and rodents) may allow for the definition of imaging surrogate marker in the future that may support the development of new causal therapeutic concepts. Computational methods excluding animal experimentation underlie many limitations in addressing the understanding of brain function. Today, translational frameworks including various models are more necessary than ever for the study of the brain’s function and dysfunction across the lifespan or the full course of the disease.

## Author Contributions

MG and JK drafted the manuscript. H-PM, FR, VR, and AL revised the manuscript for intellectual content. All the authors performed literature research, agreed to be accountable for the content of the work, and finally approved the manuscript.

## Conflict of Interest Statement

The authors declare that the research was conducted in the absence of any commercial or financial relationships that could be construed as a potential conflict of interest.
